# About Chemical Formulæ

**Published:** 1889-07-06

**Authors:** 


### Within the Wards.

### ABOUT CHEMICAL FORMULÆ.

In an equally simple, but more amusing way, Dr. Brunton discourses about the mysteries of chemical formulæ. "In discussing chemical questions," he says, "we are accustomed to use two sorts of formulæ—the 'graphic,' in which we represent diagrammatically the relative position of the atoms in a compound, and the ordinary, in which we write down its nature in symbols. In trying to convey to you an idea of chemical constitution I have used one illustration which might be compared to a graphic formula, and, in order to make the matter clearer, I may, perhaps, be allowed to use another which is more analogous to an ordinary formula. In writing down chemical formulæ we use certain letters to denote the elements, generally the initial ones of their Latin names, and the number and position of those convey to a chemist, in a certain degree, an idea of the composition of the substance represented by them. With a comparatively small number of letters of the alphabet we are able to form a great number of words, and to represent a great number of ideas." Similarly, "with a small number of (chemical) elements we can form many compounds." Just as "slight alterations in the composition of words, the introduction or abstraction of a single letter will often completely change their meaning," so "slight alterations in chemical substances will change their properties." Thus one often sees in railway carriages the announcement to passengers, "Wait until the train stops." Some mischievous fellow rubs out the T, and then it reads "Wait until the rain stops." A second lover of fun removes the lower portion of the R, and then "Wait until the pain stops" is the highly medical injunction; and the sense is as far removed from that of the original notice to passengers as it can well be. "I frequently see," continues Dr. Brunton, "an example which, I think, is common both in schools and colleges." On a certain door the words 'Class Room' were originally painted, but certain ingenious students, by obliterating the C, first turned the original word into "Lass Room," and then by removing the L degraded it to the level of "Ass Room." "Just as the successive removal of these two letters completely altered the meaning of the original word, so the removal of the letter, symbolic of two elements, from a chemical formula, will completely alter the nature of the substance represented by it." And, we may add, just as the ingenuity of the student, who by a process of elimination gradually reduced the meeting-place of himself and friends to an Ass' Parliament-house, no doubt very greatly amused both him and then, so do we heartily appreciate the idea of a congress of grave physiologists lightening their labours and enlightening their minds in this decidedly playful way.

